# Stereoselective, sex-dependent 5-HT_2A_ receptor modulation of cortical plasticity by MDMA in mice

**DOI:** 10.1038/s41386-025-02313-x

**Published:** 2026-02-02

**Authors:** Maya C. Gaines-Smith, Justin M. Silverman, Michael Fiorillo, Jason Younkin, Karah N. Moore, Jessica L. Maltman, Mario de la Fuente Revenga, Jennifer T. Wolstenholme, Richard A. Glennon, Małgorzata Dukat, Javier González-Maeso

**Affiliations:** 1 Department of Pharmacology and Toxicology, Virginia Commonwealth University School of Medicine, Richmond, VA USA; 2 Biomedical Sciences Graduate Program, Virginia Commonwealth University School of Medicine, Richmond, VA USA; 3 Virginia Institute for Psychiatric and Behavioral Genetics, Virginia Commonwealth University, Richmond, VA USA; 4 Department of Medicinal Chemistry, Virginia Commonwealth University School of Pharmacy, Richmond, VA USA; 5 Present Address: Department of Biology, Virginia State University, Petersburg, VA USA

**Keywords:** Cellular neuroscience, Neurochemistry

## Abstract

The psychoactive entactogen 3,4-methylenedioxymethamphetamine (MDMA), widely known as a recreational drug, is gaining renewed attention as a potential psychotherapeutic adjunct for treatment-resistant psychiatric disorders, yet its neurobiological mechanisms – particularly those related to its stereoisomers and sex-specific effects – remain poorly understood. Here, we report stereoselective and sex-dependent actions of MDMA on serotonin (or 5-hydroxytryptamine) 2A receptor (5-HT_2A_R)-mediated signaling and dendritic structural plasticity in mouse frontal cortex. Using both in vitro and in vivo approaches, we found that racemic MDMA and *S*(+)-MDMA exhibit weak partial agonism at 5-HT_2A_R in HEK293 cells, whereas *R*(–)-MDMA shows negligible functional activity despite higher specific binding affinity. In vivo, *S*(+)-MDMA elicited a dose-dependent head-twitch response (HTR) in both sexes, while *R*(–)-MDMA-induced HTR only in females. Correspondingly, *S*(+)-MDMA increased inositol monophosphate (IP_1_) accumulation in the frontal cortex of male and female mice, whereas *R*(–)-MDMA showed minimal effects. Structurally, *S*(+)-MDMA enhanced dendritic spine density in male frontal cortex in a partially 5-HT_2A_R-dependent manner, while no spine remodeling was observed in females or with *R*(–)-MDMA. Pharmacological blockade of the serotonin transporter (SERT) with fluoxetine fully prevented *S*(+)-MDMA-induced HTR and IP_1_ signaling, without affecting responses to the direct 5-HT_2A_R agonist DOI. These findings indicate that MDMA engages 5-HT_2A_R signaling indirectly via serotonin efflux and that this effect is both stereoselective and sex-dependent in mice, uncovering a previously unrecognized interaction between sex, MDMA stereochemistry, and 5-HT_2A_R-mediated cortical plasticity, with important implications for the rational design of MDMA-based therapeutics.

## Introduction

**Fig. 1:**
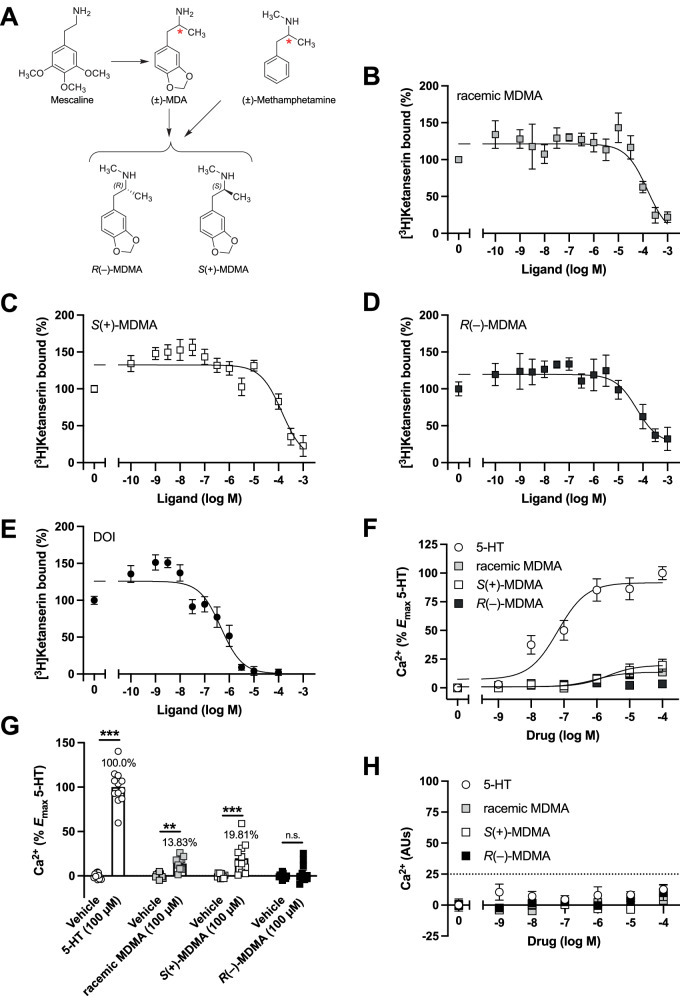
Pharmacological properties of MDMA and its enantiomers in HEK293 cells stably expressing 5-HT_2A_Rs. **A** Structural relationships among the psychedelic agent mescaline, the central stimulant methamphetamine, the psychedelic/stimulant/empathogen MDA, and the optical isomers of MDMA. Red asterisks indicate the chiral centers in the corresponding chemical structures. [^3^H]Ketanserin binding displacement curves for racemic MDMA (**B**), *S*(+)-MDMA (**C**), *R*(–)-MDMA (**D**), and DOI (**E**) in HEK293 cells stably expressing 5-HT_2A_Rs (*n* = 3–9 independent experiments performed in duplicate). **F**, **G** HEK293 cells stably expressing 5-HT_2A_Rs were loaded with Fluo-4 and monitored for [Ca^2+^]_i_ after administration of 5-HT, racemic MDMA, *S*(+)-MDMA, and *R*(–)-MDMA, or vehicle. Concentration-dependent intracellular Ca^2+^ mobilization assays (**F**) and at 100 µM (**G**) (*n* = 5–6 independent experiments performed in duplicate). **H** Concentration-dependent Ca^2+^ mobilization for 5-HT, racemic MDMA, *S*(+)-MDMA, and *R*(–)-MDMA in parental HEK293 cells (*n* = 2–4 independent experiments performed in duplicate). Statistical analysis was performed using one-way ANOVA (F[7,84] = 127.4, *p* < 0.001) followed by Bonferroni’s post-hoc test (**G**) (**F**: see Results section for F-test comparing the best fit of concentration-response curves as sigmoidal curve versus linear regression). **p < 0.01, ***p < 0.001, n.s. not significant. Data show mean ± S.E.M.

3,4-Methylenedioxymethamphetamine (MDMA) is a synthetic psychoactive compound structurally related both to the stimulant methamphetamine and the classical psychedelic mescaline [1–3]. More precisely, MDMA might be considered a hybrid of methamphetamine and the *des*-methyl counterpart of MDMA (i.e., 3,4-methylenedioxyamphetamine, or MDA – a synthetic analogue of mescaline) (Fig. 1A). Whereas mescaline is a psychedelic agent and methamphetamine is a central stimulant, MDA is a psychedelic agent with central stimulant and empathogenic actions. First synthesized and patented by Merck in 1912, MDMA remained obscure for decades until it resurfaced in the 1970s due to its distinct psychoactive profile [4]. Early clinical observations and anecdotal accounts highlighted its potential as a psychotherapeutic tool, particularly in addressing social anxiety and trauma-related conditions. Its growing popularity as a recreational drug in the early 1980s, however, led the U.S. Drug Enforcement Administration (DEA) to place it under Schedule I in 1985, hampering clinical research efforts for nearly two decades [5]. In recent years, renewed interest has led to a resurgence in clinical research, particularly in the context of posttraumatic stress disorder (PTSD) [6]. Emerging trial data suggest that MDMA may enhance therapeutic outcomes by facilitating emotional processing and reducing fear responses through mechanisms associated with fear extinction and memory reconsolidation [7–9], supporting MDMA’s classification as an empathogen/entactogen [10, 11] – terms denoting its capacity to enhance empathy and social connectedness, respectively.

Enantiomers – non-superimposable mirror images of a compound – often exhibit markedly different pharmacological profiles despite their structural similarity, a phenomenon well-documented across multiple drug classes [12–14]. For instance, among classical psychedelics, lysergic acid diethylamide (LSD) exists as a chiral compound with four isomers, yet only (+)-LSD is psychoactive [15, 16], underscoring the stereoselective or stereospecific nature of drug-receptor interactions in this class of agents. Also, as early as 1978, it was shown that whereas *R*(-)-MDA likely represents its psychedelic isomer, *S*(+)-MDA is a central stimulant [17]. Furthermore, in drug discrimination studies, it has been shown that *R*(*–*)-MDA, but not *S*(+)-MDA, substitutes in animals trained to the classical psychedelic 1-(2,5-dimethoxy-4-methylphenyl)-2-aminopropane (DOM), whereas the opposite occurs in animals trained to amphetamine; interestingly, animals trained to discriminate MDA recognized DOM, amphetamine, and both optical isomers of MDA [18]; highlighting MDA’s uniquely complex and, likely, isomer-related pharmacology. That is, the optical isomers of MDA, although producing nearly stereospecific effects, seem to share certain common actions.

MDMA also has a complex pharmacological profile, primarily due to its ability to increase levels of serotonin, as well as norepinephrine and dopamine, in the brain [19, 20]. A key mechanism involves MDMA entering the presynaptic terminal via the serotonin transporter (SERT), where it inhibits the vesicular monoamine transporter 2 (VMAT2), resulting in cytoplasmic serotonin accumulation. This is followed by a rapid and pronounced efflux of serotonin into the synaptic cleft, driven by a reversal of SERT function, thereby promoting MDMA-induced serotonin release rather than reuptake [21–23]. Although MDMA is typically used in its racemic form – comprising equal parts of the *S*(+) and *R(–)* enantiomers – few studies have directly compared the pharmacological and behavioral effects of each enantiomer. This is of particular interest because MDMA’s actions may extend beyond monoamine release, with evidence suggesting that the two enantiomers differ in their interactions with transporters and receptors, contributing to its overall pharmacodynamic complexity.

Early investigations in animal models suggested that *S*(+)-MDMA elicits greater monoamine release and stimulant-like effects, potentially conferring a high abuse liability [24], whereas *R*(–)-MDMA shows weak monoamine-releasing activity but may interact more selectively with serotonin 5-HT_2A_ receptors (5-HT_2A_Rs) [25, 26], which are G protein-coupled receptors (GPCRs) primarily coupled to heterotrimeric G_q/11_ protein-dependent signaling [27, 28]. Accordingly, other studies reported that, in drug substitution tests, cocaine more readily substitutes for *S*(+)-MDMA than for *R*(–)-MDMA in trained mice [29]. Similarly, in mice, racemic and *S*(+)-MDMA – but not *R*(–)-MDMA-induced robust hyperthermic and locomotor-stimulating effects, and pretreatment with the 5-HT_2A_R antagonist volinanserin attenuated racemic MDMA-induced hyperthermia but was less effective against *S*(+)-MDMA-induced hyperthermia, while reducing racemic MDMA- and paradoxically potentiating *S*(+)-MDMA-induced hyperlocomotion [30]. Conversely, other studies reported that certain behavioral (i.e., hyperlocomotor and hyperthermic) effects of *S*(+)-MDMA are blocked by 5-HT_2A_R antagonists in rats [31], and that the discriminative stimulus properties of cocaine resembled those of *R*(–)-MDMA rather than those of *S*(+)-MDMA when the individual MDMA optical isomers were used as training drugs [32]. However, the 5-HT_2_R antagonists ketanserin and pirenperone failed to block the discriminative stimulus effects of MDMA [33] – underscoring the complex interplay between monoamine release and receptor-mediated signaling. Reflecting this complexity – while *S*(+)-MDMA-induced subjective “intoxication” in healthy human volunteers at lower doses than either racemic MDMA or *R*(-)-MDMA [4], both rodent [34] and human [35] studies suggest that the actions of individual MDMA isomers might not reflect the overall actions of racemic MDMA. In line with this, recent head-to-head comparisons in healthy participants indicate that neither enantiomer alone confers a clear therapeutic advantage over the racemic mixture in the context of substance-assisted therapy [36]. These findings underscore the need for further mechanistic research to clarify the differential actions of each MDMA isomer, as their unique effects may vary depending on the species studied, experimental paradigm, or dosage, and may not be fully captured by the pharmacology of the racemic formulation alone.

Atrophy of frontal cortex neurons has been implicated in the pathophysiology of several psychiatric disorders [37, 38]. In rodent models, a single administration of psychedelics such as psilocybin, 5-methoxydimethyltryptamine (5-MeO-DMT), or 1-(2,5-dimethoxy-4-iodophenyl)-2-aminopropane (DOI) promotes rapid dendritic structural plasticity in frontal cortex pyramidal neurons – a process largely mediated by the 5-HT_2A_R [37, 39, 40]. Similar neuroplastic effects have been observed with ketamine [41, 42]. However, whether MDMA – or its enantiomers – induces comparable structural remodeling in the frontal cortex, and to what extent serotonin GPCRs, including the 5-HT_2A_R, contribute to its therapeutically relevant effects – either directly via orthosteric agonism or indirectly through SERT-mediated mechanisms – in a potentially stereoselective manner, remains unclear.

In the present study, we investigated the post-acute effects of a single administration of either *S*(+)-MDMA or *R*(–)-MDMA on head-twitch response (HTR) (a rodent behavioral proxy of 5-HT_2A_R-mediated human psychedelic activity [43]), inositol monophosphate (IP_1_) accumulation (a downstream marker of G_q/11_ signaling) [44], and dendritic structural plasticity in the frontal cortex of male and female mice. Our findings reveal, for the first time, stereoselective and sex-specific synaptic and behavioral plasticity effects of MDMA that are at least partially mediated indirectly via the 5-HT_2A_R through MDMA-induced serotonin release in the frontal cortex.

## Materials and methods

### Drugs

(±)-1-(2,5-Dimethoxy-4-iodophenyl)-2-aminopropane (DOI) hydrochloride, 5-hydroxytryptamine (5-HT, or serotonin) hydrochloride, fluoxetine hydrochloride, para-chlorophenylalanine (PCPA) and volinanserin (M100907) were purchased from Sigma-Aldrich (St. Louis, MO). Methysergide maleate was purchased from Tocris (Minneapolis, MN). For radioligand binding assays, [^3^H]ketanserin was obtained from PerkinElmer (Shelton, CT). 3,4-Methylenedioxymethamphetamine [also known as N-methyl-1-(3,4-methylenedioxyphenyl)-2-aminopropane] (MDMA), and both its optical isomers as their hydrochloride salts were gifts from the National Institute on Drug Abuse (NIDA). All other chemicals were purchased from standard sources.

### Mammalian cell lines

Human embryonic kidney cells (HEK293 cells) were purchased from American Type Culture Collection (Manassas, VA) (CRL-1573). HEK293 cells stably expressing human 5-HT_2A_R have been described previously [45]. Cells were maintained in Dulbecco’s modified Eagle’s medium supplemented with 10% (v/v) dialyzed FBS and 1% penicillin/streptomycin (Thermo Fisher Scientific, Walthman, MA) in a 5% CO_2_ humidified atmosphere at 37 °C.

### Radioligand binding assays

Radioligand binding assays were performed as previously reported [45], with minor modifications. Briefly, cells were homogenized in Tris-HCl buffer, followed by sequential centrifugation to isolate membrane (P_2_) fractions. Binding reactions were performed at 37 °C for 60 min using [^3^H]ketanserin (5 nM) and varying concentrations of test compounds. Non-specific binding was defined using methysergide (10 µM). Bound and free ligand were separated by vacuum filtration using a MicroBeta Filtermat-96 harvester (PerkinElmer, Shelton, CT), and radioactivity was quantified via liquid scintillation using a MicroBeta2 detector (PerkinElmer, Shelton, CT).

### Intracellular calcium mobilization

Intracellular calcium mobilization assays were performed as previously reported [45], with minor modifications. Briefly, cells plated on poly-D-lysine–coated 96-well plates were loaded with Fluo 4-AM in imaging buffer prior to testing. Fluorescence (excitation: 494 nm; emission: 525 nm) was measured using a FlexStation 3 system following compound addition. Data were analyzed using SoftMax Pro software (Molecular Devices, Wokingham, UK).

### Animals and drug administration

Experiments were performed on adult (8-17 week-old) C57BL/6J (The Jackson Laboratory, Bar Harbor, ME) male and female mice. For assays with *5-HT*_*2A*_*R* (*Htr2a*) knockout mice (*5-HT*_*2A*_*R-KO*) [43], heterozygous mice on a C57BL/6J background were bred to obtain *5-HT*_*2A*_*R-WT* and *5-HT*_*2A*_*R-KO* mice, and confirmed by genotyping tail snips. Animals were housed in groups of 3–5 littermates per cage under a 12 h light/dark cycle at 23 °C, with food and water *ad libitum*, except during behavioral testing. Experiments were conducted in accordance with NIH guidelines, and were approved by the Virginia Commonwealth University Animal Care and Use Committee. All efforts were made to minimize animal suffering and the number of animals used.

### Head twitch response

Detection of head-twitch responses (HTR) in mice was performed as previously reported [46, 47]. Briefly, mice were ear-tagged with neodymium magnets (N50, 3 mm diameter × 1 mm height, 50 mg) glued to the top surface of aluminum ear tags for rodents (Las Pias Ear Tag, Stoelting Co.) with the magnetic south of the magnet in contact with the tag. Following ear-tagging animals were placed back into their home cages and allowed to become accustomed to the tags for one week. As shown previously [47], although mild reddening of the ear was occasionally observed, the magnetic tags were generally well tolerated by the animals, even when housed in groups. On test days, mice were placed individually into the monitoring chamber for 15 min to acclimate to the environment and determine baseline HTR. Subsequently, the animals received (i.p.) the corresponding dose of *S*(+)-MDMA, *R*(–)-MDMA or DOI, or vehicle, and HTRs were recorded for 60 min. This dose range was selected because higher doses of MDMA have been shown to induce detrimental effects on fear learning and memory [48]. For antagonist testing, mice received volinanserin (0.1 mg/kg) 15 min prior to the administration of the tested compound (or vehicle).

### IP_1_ experiment

The non-radioactive method to capture changes in inositol monophosphate (IP_1_) in mouse frontal cortex samples was performed as previously reported [44], with minor modifications. Briefly, mice were sacrificed one hour after receiving *S*(+)-MDMA, *R*(–)-MDMA, DOI, or vehicle (i.p). This time point corresponds approximately to the half-life of MDMA in rat plasma [49]. Frontal cortices (bregma 1.9 to 1.40 mm) were collected, homogenized in lysis buffer, and centrifuged. Clarified supernatants were analyzed using an HTRF-based IP-One Gq assay in 96-well plates. IP_1_ levels were quantified by measuring fluorescence emission ratios (615/665 nm) using a VICTOR Nivo plate reader from PerkinElmer (Shelton, CT), and results were normalized to vehicle-treated controls. A standard curve (0–1.1 μM IP_1_) confirmed assay linearity within the detection range.

### Dendritic spine density

Stereotaxic surgery [50–52] and dendritic spine density assays [40, 53] were performed as previously described, with minor modifications. Briefly, mice were anesthetized with isoflurane, secured in a stereotaxic frame, and prepared for surgery by making a midline incision along the dorsal surface of the skull. The resulting skin flaps were retracted and held in place with bulldog clamps (Fine Science Tools). Bregma was identified, and bilateral injection coordinates targeting the prelimbic region of the medial prefrontal cortex the mouse brain were determined relative to bregma (+1.6 mm rostrocaudal, ±2.6 mm mediolateral, −2.0 mm dorsoventral). The coordinates were taken according to a published atlas of the mouse C57BL/6 strain [54]. Bilateral craniotomies were made using a Micro-Drill™ (Ideal), and residual blood was gently cleared with sterile cotton swabs. Hamilton syringes were loaded with 1 µl (1:1 dilution with PBS) of HSV-GFP (NeuroTools, Chapel Hill, NC) and positioned bilaterally at a 10° angle. Syringes were lowered into the target sites at a rate of 0.5 mm/min, and injections were performed at 0.1 µl/min. This bicistronic p10051+ replication-deficient HSV-1 amplicon vector expresses GFP under the control of the CMV promoter and was produced using a helper-free packaging system, ensuring both biosafety (BSL-1) and negligible neurotoxicity [55]. After recovery, mice received intraperitoneal (i.p.) injections of *S*(+)-MDMA (3 mg/kg), *R*(–)-MDMA (3 mg/kg), or vehicle. Twenty-four hours later, mice were transcardially perfused, and brains were fixed. Whole brains were then collected and post-fixed in 4% PFA at 4 °C for 24 h, followed by storage in PBS at 4 °C until sectioning at 30 µm. For spine analysis, dendrites (apical and basal) were randomly selected from GFP-labeled pyramidal neurons, which were identified by their characteristic triangular soma. Dendritic segments selected for analysis met the following criteria: (i) the segment was completely filled (endings were excluded), and (ii) the segment could be traced directly back to the soma. Images of pyramidal neurons in layer V of the frontal cortex were acquired using confocal microscopy (40× and 63×, Z-stacks), and dendritic spines were manually counted by a blinded scorer in 20-µm segments.

### Tissue sample collection

The day of the experiment, mice were sacrificed for analysis by cervical dislocation, and bilateral frontal cortex (bregma 1.9 to 1.40 mm) were dissected, and either frozen at −80 °C, or immediately processed for biochemical assays.

### Enzyme-linked immunosorbent assay (ELISA)

Mice received (i.p.) PCPA (100 mg/kg) once daily for four consecutive days, and frontal cortex samples were collected on the fifth day. Frontal cortex tissue was homogenized in ice-cold PBS at a 1:9 (w/v) ratio, followed by centrifugation at 5000 × *g* for 10 min at 4 °C. The resulting supernatants were collected and stored at −80 °C until analysis. 5-HT levels were measured using a commercially available ELISA kit (E-EL-0033, Elabscience) according to the manufacturer’s instructions. All samples were run in duplicate, and absorbance was measured at 450 nm using a VICTOR Nivo multimode microplate reader (PerkinElmer, Shelton, CT) to generate a standard curve, from which protein concentrations were calculated. VICTOR Nivo plate reader from PerkinElmer (Shelton, CT),

### Statistical analysis

Statistical significance was assessed by Student’s *t* test, and one-way, nested one-way, two-way or three-way ANOVA, depending upon the number of experimental conditions and independent variables. When ANOVA indicated a significant effect, post-hoc comparisons were performed using a Bonferroni’s test. K_i_ values were calculated from IC_50_ values using the Cheng–Prusoff equation [56], with a previously determined [^3^H]ketanserin K_D_ of 0.805 nM [45]. An extra sum-of-squares F-test was used to determine whether a sigmoidal concentration-response curve or a straight line provided the better fit to the data. Animals were randomly assigned to experimental groups. Statistical power and sample size estimates were calculated assuming β = 0.20 (80% power) and a type I error rate of 5%, with biologically meaningful differences between means defined based on our prior experience with similar protocols. For behavior and dendritic spine density assays, experimenters were blinded to the treatment and genotype groups. Data points were excluded based on a pre-established criterion of ±2 standard deviations from the group mean. All values are expressed either as mean ± S.E.M., or as box plots showing, in ascending order, the minimum value, first quartile, median, third quartile, and maximum value. Statistical analyses were conducted using GraphPad Prism version 10, and results were considered significant at *p* < 0.05.

## Results

### 5-HT_2A_R-dependent pharmacological properties of MDMA and its enantiomers in HEK293 cells

[^3^H]Ketanserin binding displacement assays were performed in HEK293 cells stably expressing the human 5-HT_2A_R. As expected, based on previous studies [20, 57, 58], racemic MDMA displaced [^3^H]ketanserin binding with moderate affinity in the low micromolar range (pK_i_ = 4.71 ± 0.18 (Fig. 1B). Additionally, *S*(+)-MDMA (Fig. 1C) and R(–)-MDMA (Fig. 1D) displayed lower (pK_i_ = 4.43 ± 0.28) and higher (pK_i_ = 4.89 ± 0.32) affinities, respectively, compared to the racemic mixture. As previously reported [43], DOI (Fig. 1E) displaced [^3^H]ketanserin with a much higher affinity (pK_i_ = 7.17 ± 0.22). To assess agonist activity at the 5-HT_2A_R, functional assays revealed that both racemic MDMA (E_max_ = 13.69 ± 1.92; pE_50_ = 6.03 ± 0.36) and *S*(+)-MDMA (E_max_ = 19.72 ± 2.75; pE_50_ = 5.68 ± 0.37) exhibited weak agonist properties, with their concentration-response curves better fit by a sigmoidal curve than by linear regression (F[1,81] = 4.57, *p* < 0.05 and *F*[1,62] = 5.42, *p* < 0.05, respectively) (Fig. 1F). In contrast, *R*(–)-MDMA did not elicit a measurable response relative to the full agonist 5-HT (E_max_ = 91.55 ± 4.42; pE_50_ = 7.18 ± 0.18), and its data were better fit by a linear regression than by a sigmoidal curve (F[1,80] = 0.77, *p* > 0.05) (Fig. 1F). When intracellular calcium release was evaluated at a single concentration (100 µM), both racemic and *S*(+)-MDMA produced significant effects relative to vehicle control (Fig. 1G). In comparison, the response to *R*(–)-MDMA (100 µM) was indistinguishable from vehicle, with 5-HT (100 µM) serving as a positive control (Fig. 1G). As an additional internal control, no calcium response was observed in parental (non-transfected) HEK293 cells exposed to racemic MDMA, *S*(+)-MDMA, *R*(–)-MDMA, or 5-HT (Fig. 1H).

### HTR induced by MDMA and its enantiomers in male and female mice

**Fig. 2:**
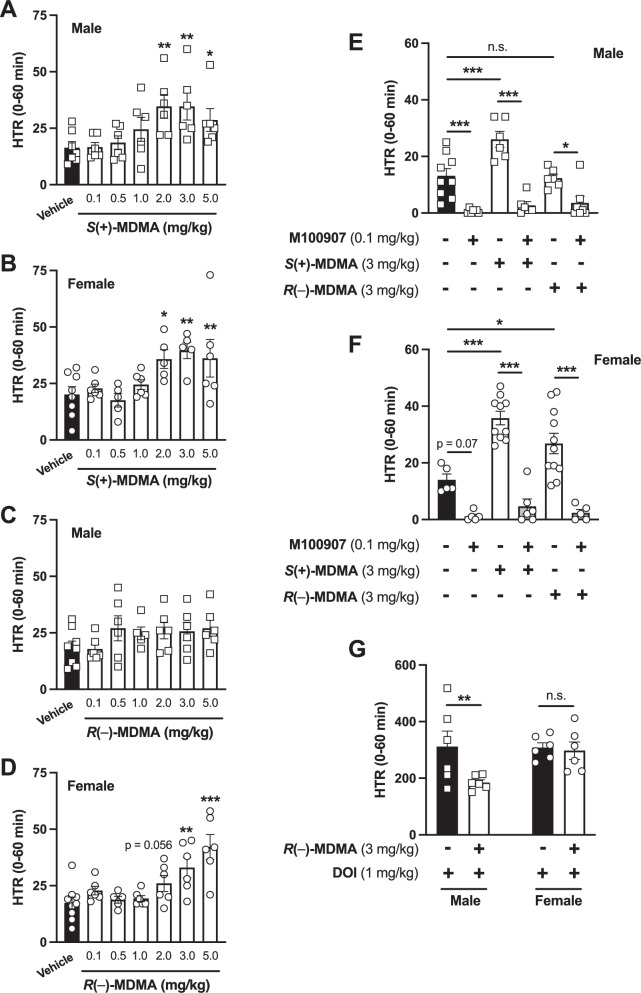
Effect of *S*(+)-MDMA, and *R*(–)-MDMA on HTR. Dose-response effect of *S*(+)-MDMA (**A**, **B**), and *R*(–)-MDMA (**C**, **D**) on HTR in male (**A**, **C**) and female (**B**, **D**) mice. HTR counts correspond to the first 60 min after injection (i.p.) with the indicated dose of *S*(+)-MDMA or *R*(–)-MDMA, or vehicle in male (*n* = 5–8 per group) and female (*n* = 5–9 per group) mice (**A**: *F*[6,36] = 3.38, *p* < 0.001; **B**: *F*[6,34] = 3.95, *p* < 0.01; **C**: *F*[6,36] = 1.24, *p* > 0.05; **D**: *F*[6,37] = 7.39, *p* < 0.001). **E**, **F** Effect of pretreatment (i.p.) with the 5-HT_2A_R antagonist volinanserin (M100907) (0.1 mg/kg) or vehicle 15 min prior to the administration (i.p.) of *S*(+)-MDMA (3 mg/kg) and *R*(–)-MDMA (3 mg/kg), or vehicle on HTR in male (*n* = 6–9 per group) (**E**) and female (*n* = 5–11 per group) (**F**) mice (**E**: MDMA isomer *F*[2,35] = 7.07, *p* < 0.01; M100907 *F*[1,35] = 74.40, *p* < 0.001; interaction *F*[2,35] = 5.95, *p* < 0.01. **F** MDMA isomer *F*[2,36] = 7.64, *p* < 0.01; M100907 *F*[1,36] = 77.63, *p* < 0.001; interaction *F*[2,36] = 4.01, *p* < 0.05). **G** Effect of pretreatment (i.p.) with *R*(–)-MDMA (3 mg/kg), or vehicle 15 min prior to the administration (i.p.) of DOI (1 mg/kg), or vehicle on HTR in male (*n* = 6 per group) and female (*n* = 6 per group) mice (drug *F*[1,20] = 6.44, *p* < 0.05; sex *F*[1,20] = 4.42, *p* < 0.05; interaction *F*[1,20] = 4.88, *p* < 0.05). Statistical analysis was performed using one-way (**A**–**D**) or two-way (**E**–**G**) ANOVA followed by Bonferroni’s post-hoc test. (**A**–**D**: see Results section for two-way ANOVA analysis evaluating main effects of drug and sex, as well as their interaction) (**E** and **F**: see Supplementary Table 1 for three-way ANOVA analysis evaluating main effects of MDMA isomer, M100907 and sex, as well as their interaction). *p < 0.05, **p < 0.01, ***p < 0.001, n.s. not significant. Data show mean ± S.E.M.

Compared to the effect of classical psychedelic 5-HT_2A_R agonists such as DOI [59] (see also below), our data indicate that *S*(+)-MDMA produced a moderate, yet statistically significant, dose-dependent HTR in both male (Fig. 2A) and female (Fig. 2B) mice. In contrast, *R*(–)-MDMA induced a dose-dependent HTR only in female animals, with male mice showing no response at any of the tested doses (Fig. 2C, D). Two-way ANOVA indicated only a dose effect – and no sex or interaction effects – for *S*(+)-MDMA (dose: *F*[6,70] = 7.07, *p* < 0.001; sex: *F*[1,70] = 1.82, *p* > 0.05; interaction: *F*[6,70] = 0.26, *p* > 0.05), whereas significant dose and interaction effects, but not a sex effect, were found for the *R*(–)-MDMA enantiomer (dose: *F*[6,73] = 5.62, *p* < 0.001; sex: *F*[1,73] = 0.91, *p* > 0.05; interaction: *F*[6,73] = 2.46, *p* < 0.05).

To assess the involvement of the 5-HT_2A_R in these effects, a separate cohort of mice was pretreated with the 5-HT_2A_R antagonist volinanserin (0.1 mg/kg) prior to administration of *S*(+)-MDMA (3 mg/kg), *R*(–)-MDMA (3 mg/kg), or vehicle (Fig. 2E, F). Analysis of the cumulative 60-min HTR revealed that volinanserin pretreatment significantly reduced the HTR induced by both *S*(+)-MDMA and *R*(–)-MDMA in female mice (Fig. 2F). In males, volinanserin also significantly attenuated the *S*(+)-MDMA-induced HTR, whereas, consistent with the data shown in Fig. 2C, R(–)-MDMA did not elicit HTR in male mice (Fig. 2E). Volinanserin administered alone produced a significant – or trend-level – reduction in HTR compared with vehicle-treated animals in males (Fig. 2E) and females (Fig. 2F), respectively, and significantly reduced HTR relative to *R*(–)-MDMA-treated male mice (Fig. 2E). A three-way ANOVA revealed significant main effects of MDMA isomer, volinanserin, and sex, as well as significant interactions among some of these factors (Supplementary Table 1).

### *R*(–)-MDMA reduces DOI-induced HTR in male, but not female, mice

Considering that *R*(–)-MDMA lacked agonistic activity at the canonical pathway downstream of the 5-HT_2A_R in HEK293 cells (Fig. 1F, G), yet induced sex-biased HTR characterized by an effect in female (Fig. 2D) but not in male mice (Fig. 2C), we tested whether this isomeric form of MDMA could allosterically modulate the HTR induced by the classical psychedelic DOI in either sex. Interestingly, preadministration of *R*(–)-MDMA (3 mg/kg) reduced DOI (1 mg/kg)-induced HTR in male, but not in female, mice (Fig. 2G).

### IP_1_ accumulation induced by MDMA and its enantiomers the frontal cortex of male and female mice

**Fig. 3:**
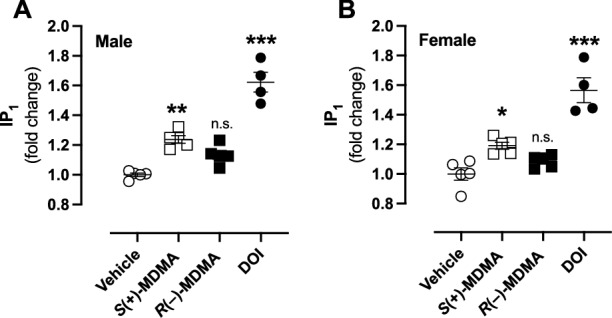
Effect of *S*(+)-MDMA, and *R*(–)-MDMA on IP_1_ accumulation in the frontal cortex. Samples were collected 60 min after administration (i.p.) of *S*(+)-MDMA (3 mg/kg), *R*(–)-MDMA (3 mg/kg) and DOI (5 mg/kg), or vehicle in male (**A**) and female (**B**) mice (*n* = 4–5 per group) (**A**: *F*[3,15] = 52.98, *p* < 0.001; **B** *F*[3,15] = 29.04, *p* < 0.001). Statistical analysis was performed using one-way ANOVA followed by Bonferroni’s post-hoc test (**A**, **B**). (**A**, **B**: see Results section for two-way ANOVA analysis evaluating main effects of drug and sex, as well as their interaction). *p < 0.05, **p < 0.01, ***p < 0.001, n.s. not significant. Data show mean ± S.E.M.

To further evaluate potential sex differences in 5-HT_2A_R-dependent signaling, we assessed the accumulation of IP_1_, a downstream effector of the G_q/11_ signaling pathway, in vivo. As expected, based on prior studies with the orthosteric 5-HT_2A_R agonist [44], DOI (5 mg/kg)-treated animals showed robust IP_1_ accumulation in the frontal cortex in both male (Fig. 3A) and female (Fig. 3B) mice. Administration of *S*(+)-MDMA (3 mg/kg) also induced IP_1_ accumulation in the frontal cortex of both sexes (Fig. 3A, B). In contrast, *R*(–)-MDMA (3 mg/kg) produced a trend toward increased IP_1_ accumulation in male mice (*p* = 0.08) (Fig. 3A), but had no detectable effect in their female counterparts (*p* = 0.51) (Fig. 3B). Two-way ANOVA indicated significant a drug effect, with no significant effects of sex or drug × sex interaction (drug: *F*[3,30] = 76.5, *p* < 0.001; sex: *F*[1,30] = 1.79, *p* = 0.19; interaction: *F*[3,30] = 0.21, *p* > 0.05).

### Sex-specific effects of MDMA isomers on frontal cortex dendritic spine density are partially mediated via 5-HT_2A_R

**Fig. 4:**
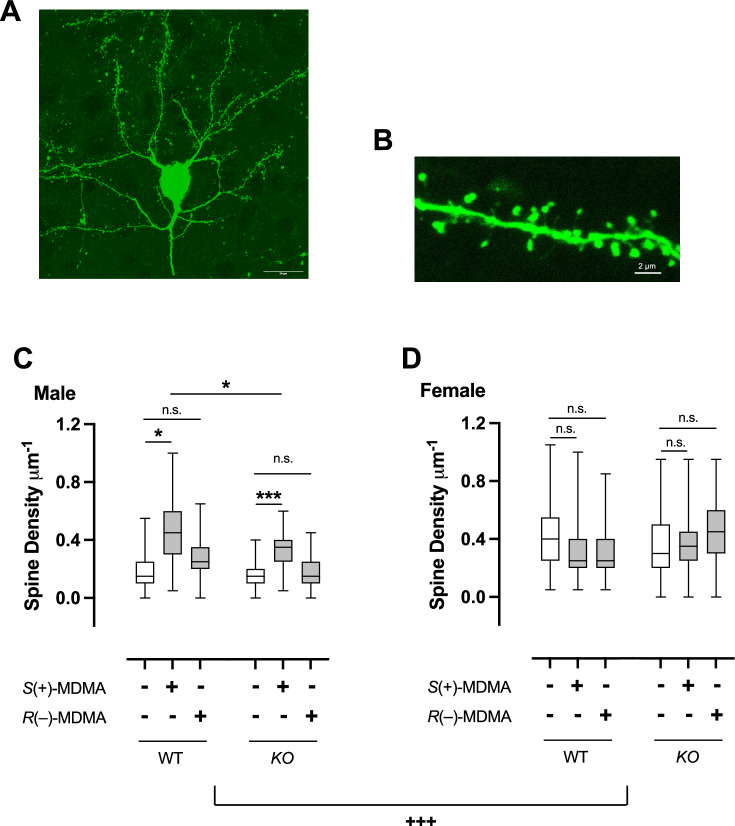
Sex-specific post-acute effect of *S*(+)-MDMA, and *R*(–)-MDMA on dendritic spine density in the frontal cortex of *5-HT*_*2A*_*R-KO* mice and wild-type controls. **A**–**F** Samples were collected 24-h after a single injection (i.p.) of *S*(+)-MDMA (3 mg/kg), and *R*(–)-MDMA (3 mg/kg) or vehicle. **A** Representative image of HSV-mediated transgene expression in the frontal cortex. HSV-GFP was injected in the frontal, and GFP expression was revealed by fluorescence microscopy imaging. **B** Representative three-dimensional reconstructions of HSV-injected frontal cortex dendritic segments. **C** Dendritic spine density in male wild-type (*n* = 11–37 neurons from 3 to 4 mice) and *5-HT*_*2A*_*R-KO* (*n* = 30–119 neurons from 2 to 4 mice) animals (WT: *F*[2,8] = 4.84 *p* < 0.05; *KO*: *F*[2,7] = 34.16 *p* < 0.001). **D** Dendritic spine density in female wild-type (*n* = 12–48 neurons from 3 to 4 mice) and *5-HT*_*2A*_*R-KO* (*n* = 25–99 neurons from 2 to 4 mice) animals (WT: *F*[2,7] = 0.35 *p* > 0.05; *KO*: *F*[2,6] = 0.97 *p* > 0.05). Scale bars represent 20 µm (**A**). Statistical analysis was performed using nested one-way or three-way (^+++^*p* < 0.001) ANOVA followed by Bonferroni’s multiple comparison test (*p < 0.05, ***p < 0.001, n.s. not significant). (**C**, **D**: see Supplementary Table 2 for additional three-way ANOVA analysis evaluating main effects of MDMA isomer, genotype and sex, as well as their interaction). Box plots in (**C**) and (**D**) present, in ascending order, the minimum value, first quartile, median, third quartile and maximum value of the sample data, expressed as spines per µm.

We next examined the post-acute effects (24-h post-administration) of *S*(+)-MDMA and *R*(–)-MDMA on dendritic spine density in the frontal cortex of *5-HT*_*2A*_*R-KO* mice and their control littermates of both sexes. Mice received stereotaxic injections of an HSV-GFP viral vector into the frontal cortex, which resulted in robust GFP expression within the targeted region (Fig. 4A). Following a 3-day recovery period, animals were administered a single dose of *S*(+)-MDMA (3 mg/kg), *R*(–)-MDMA (3 mg/kg), or vehicle, and brain tissue was collected 24-h later (Fig. 4B). Baseline comparisons revealed a higher dendritic spine density in the frontal cortex of vehicle-treated female mice compared to males (Supplementary Fig. 1). Interestingly, in wild-type male mice, *S*(+)-MDMA significantly increased dendritic spine density in the frontal cortex (Fig. 4C) – an effect that was attenuated, yet still detectable, in *5-HT*_*2A*_*R-KO* littermates (Fig. 4C). In contrast, *R*(–)-MDMA did not alter dendritic spine density in either genotype within the male cohort (Fig. 4C). Female mice, regardless of genotype, exhibited no post-acute changes in dendritic spine density following treatment with either enantiomer (Fig. 4D). A three-way ANOVA revealed significant main effects of drug, genotype, and sex, as well as significant interactions among these factors (Supplementary Table 2).

### HTR and IP_1_ accumulation in the frontal cortex induced by MDMA isomers are prevented by pharmacological blockade of the serotonin transporter

Our next goal was to evaluate whether preventing serotonin efflux would reduce MDMA-induced behavior and 5-HT_2A_R-dependent signaling. One potential pharmacological approach to achieve this is the use of para-chlorophenylalanine (PCPA), a selective and irreversible inhibitor of tryptophan hydroxylase – the rate-limiting enzyme in serotonin biosynthesis [60]. To assess the extent to which repeated PCPA administration reduces serotonin content, mice received PCPA (100 mg/kg) once daily for four consecutive days, and frontal cortex samples were collected on the fifth day. ELISA-based quantification revealed that PCPA significantly reduced frontal cortex 5-HT levels in a sex-specific manner. In male mice, this reduction was only partial (~50% compared to vehicle-treated animals) (Supplementary Fig. 2), consistent with previous microdialysis findings in mouse frontal cortex [61]. However, 5-HT levels in the frontal cortex of female mice were unaffected by the same regimen of PCPA administration (Supplementary Fig. 2).

**Fig. 5:**
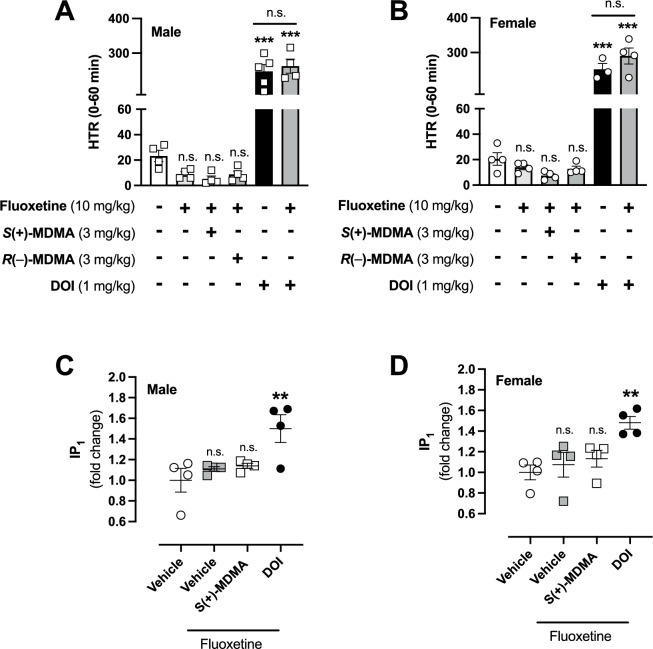
Effect fluoxetine on HTR and frontal cortex IP_1_ accumulation induced by *S*(+)-MDMA, and *R*(–)-MDMA in male and female mice. **A**, **B** Effect of pretreatment (i.p.) with fluoxetine (10 mg/kg) or vehicle 60 min prior to the administration (i.p.) of *S*(+)-MDMA (3 mg/kg), *R*(–)-MDMA (3 mg/kg) and DOI (1 mg/kg), or vehicle on HTR in male (**A**) and female (**B**) mice (*n* = 3–5 per group) (**A**: *F*[5,19] = 98.96, *p* < 0.001; **B** *F*[5,17] = 132.7, *p* < 0.001). **C**, **D** Effect of pretreatment fluoxetine (10 mg/kg, i.p.) or vehicle, administered 60 min prior to *S*(+)-MDMA (3 mg/kg, i.p.), DOI (5 mg/kg, i.p.), or vehicle, on IP_1_ accumulation in the frontal cortex of male (**C**) and female (**D**) mice (*n* = 4 per group). Samples were collected 60 min after administration of *S*(+)-MDMA, DOI, or vehicle. (**C**: *F*[3,12] = 5.81, *p* < 0.05; **D**: *F*[3,12] = 6.10, *p* < 0.01). Statistical analysis was performed using one-way ANOVA followed by Bonferroni’s post-hoc test (**A**–**D**). **p < 0.01, ***p < 0.001, n.s. not significant. Data show mean ± S.E.M.

Due to this incomplete and sex-biased depletion of serotonin, we next employed an alternative strategy to block SERT using fluoxetine, a selective serotonin reuptake inhibitor that binds to the central site of the transporter and prevents serotonin reuptake directly [62]. Notably, administration of fluoxetine (10 mg/kg) 60-min prior to *S*(+)-MDMA (3 mg/kg) completely abolished the HTR in both male (Fig. 5A) and female (Fig. 5B) mice. Similarly, fluoxetine pretreatment fully prevented *R*(–)-MDMA (3 mg/kg)-induced HTR in female mice (Fig. 5B), while having no effect on the absence of HTR induced by this MDMA enantiomer in male counterparts (Fig. 5A). Furthermore, fluoxetine (10 mg/kg) pretreatment did not alter DOI-induced HTR in either male (Fig. 5A) or female (Fig. 5B) animals. A similar effect was observed when evaluating IP_1_ accumulation in the frontal cortex. Specifically, fluoxetine (10 mg/kg) administration fully prevented the effect of *S*(+)-MDMA, but not DOI, on IP_1_ accumulation in both male (Fig. 5C) and female mice (Fig. 5C). Two-way ANOVA indicated significant a drug effect, with no significant effects of sex or drug × sex interaction (Supplementary Fig. 3).

## Discussion

This study presents the first comprehensive characterization of the stereoselective and sex-specific effects of MDMA on behavior and synaptic structural plasticity in mouse frontal cortex, implicating indirect partial activation of 5-HT_2A_R via SERT-mediated serotonin efflux as a key mechanism underlying these effects. We show that *S*(+)- and *R*(–)-MDMA display functionally distinct in vitro profiles and elicit divergent, sex-dependent outcomes in vivo, including differences in intracellular signaling, dendritic spine remodeling, and 5-HT_2A_R-mediated behaviors.

Pharmacological profiling in HEK293 cells revealed that *S*(+)-MDMA exhibits weak partial agonism at the 5-HT_2A_R, whereas *R*(–)-MDMA displays negligible intrinsic activity despite displacing [^3^H]ketanserin binding with higher affinity than its optical isomer. In classical pharmacology, and in line with the extended ternary complex model [63, 64], orthosteric agonists often exhibit lower affinity for a given GPCR target compared to structurally similar orthosteric antagonists. Our findings replicate previous observations reporting that the *S(+)* enantiomer has lower affinity for the 5-HT_2A_R than its *R(–)* counterpart [57, 58]. Additionally, these binding results seem to be consistent with our functional assays, which showed that only *S*(+)-MDMA-induced 5-HT_2A_R-dependent intracellular Ca^2+^ release in HEK293 cells, whereas *R*(–)-MDMA did not elicit such a response.

While previous studies [20], as well as ours, suggest that racemic MDMA acts as a weak partial agonist at the 5-HT_2A_R, to our knowledge, only one prior report has assessed the intrinsic activity of MDMA enantiomers at this receptor in cell-based assays. In contrast to our findings, that study reported that *R*(–)-MDMA exhibited greater efficacy in activating 5-HT_2A_R-mediated accumulation of [^3^H]inositol monophosphate [25]. Interestingly, the same study found the opposite pattern of efficacy at the 5-HT_2C_R, with *S*(+)-MDMA showing greater activity than *R(–)-MDMA* [25], along with a similar pharmacological profile at the 5-HT_2B_R, as reported in a follow-up study [65]. Although further investigation is clearly needed to reconcile these differing results, such discrepancies may reflect differences in assay readouts (e.g., G_q/11_-coupled second messenger accumulation versus calcium flux) or in transgene expression levels between stably expressing cell lines including human embryonic kidney (HEK293) and mouse fibroblast (3T3) cells.

In vivo, both MDMA enantiomers induced HTR in female mice, whereas only *S*(+)-MDMA elicited this response in males. Pretreatment with the 5-HT_2A_R antagonist volinanserin completely abolished these effects, confirming that orthosteric activation of 5-HT_2A_R is required for HTR induction by both enantiomers. The absence of HTR following *R*(–)-MDMA in males, together with IP_1_ accumulation assays showing that *S*(+)-MDMA robustly increased G_q/11_-mediated signaling in the frontal cortex of both sexes – whereas *R*(–)-MDMA failed to do so in either – supports the interpretation that *R*(–)-MDMA functions as an inefficient direct 5-HT_2A_R non-agonist, at least in males. However, given that *R*(–)-MDMA attenuated DOI-induced HTR in males but not in females, and that fluoxetine pretreatment reduced the HTR elicited by both *S*(+)- and *R*(–)-MDMA in females, the data collectively suggest that *R*(–)-MDMA may act as a weak 5-HT_2A_R antagonist in males, while in females it induces HTR through 5-HT_2A_R activation that depends on 5-HT release via SERT. Furthermore, because *R*(–)-MDMA failed to increase IP_1_ accumulation in the frontal cortex of female mice, the observed HTR likely involves signaling pathways distinct from the canonical G_q/11_-IP_3_-IP_1_ cascade, potentially engaging PTX-sensitive G_i/o_ proteins [43], β-arrestin-dependent mechanisms [66], or other GPCRs not antagonized by volinanserin but acting in concert with 5-HT_2A_R to mediate this female-selective response.

One of the most striking findings of this study is the sex-dependent, stereospecific modulation of dendritic spine density. Only the *S*(+)-MDMA enantiomer increased spine density in the frontal cortex of male mice, whereas neither enantiomer had an effect in females. This structural plasticity effect was partially attenuated in *5-HT*_*2A*_*R-KO* mice, indicating 5-HT_2A_R signaling contributes – at least in part – to the changes induced by *S*(+)-MDMA in males. These findings parallel and extend recent studies showing that psychedelics and other neuroplasticity-promoting compounds induce rapid spinogenesis in frontal cortex pyramidal neurons through 5-HT_2A_R-dependent mechanisms [37, 39, 40, 67]. Our findings suggest that MDMA similarly engages this serotonergic pathway – but with sex-specific divergences. It remains to be determined whether the weak direct agonist activity of *S*(+)-MDMA at the 5-HT_2A_R, together with its indirect activation through 5-HT via SERT reversal, contributes to its greater efficacy in promoting frontal cortex structural plasticity and HTR – relative to the *R*(–)-MDMA enantiomer – in a sex-dependent manner. The mechanistic basis for the sex-specific differences observed between *S*(+)- and *R*(–)-MDMA – as well as the sex-related differences in frontal cortex dendritic spine density in vehicle-treated animals – remains unclear, but may involve variations in serotonergic tone, estrogen signaling, or downstream transcriptional responses, all of which are known to modulate neuroplasticity [59, 68, 69]. An important question for further investigation is whether the higher frontal cortex dendritic spine density observed in female mice relative to males under control conditions (i.e., vehicle-treated animals) represents a ceiling effect that constrains additional increases following *S*(+)- and/or *R*(–)-MDMA administration. From a broader perspective, although the dose range of *S*(+)-MDMA and *R*(–)-MDMA used here was based on previous findings indicating that higher doses of MDMA impair behavioral plasticity [48], whether increasing the dose of either enantiomer or collecting samples at different time points differentially affects plasticity processes in the frontal cortex – or alternatively, in subcortical regions such as the nucleus accumbens – remains an important question for future studies.

Regarding the isomer-specific abuse potential of MDMA, several studies have examined cocaine substitution in drug discrimination paradigms. Some reports indicate that the behavioral effects of cocaine more closely resemble those of *R*(–)-MDMA than *S*(+)-MDMA in rats [32]. In other studies, cocaine substituted for both *S*(+)-MDMA and *R*(–)-MDMA, with greater potency observed in mice trained to recognize *S*(+)-MDMA [29]. Moreover, the psychedelic 5-HT_2A_R agonists 1-(2,5-dimethoxy-4-methylphenyl)-2-aminopropane (DOM) [70] and α-methyltryptamine (α-MeT) [71] fail to substitute for MDMA in similar paradigms. Sex- and enantiomer-dependent differences have also been observed in substitution tests using rats trained to discriminate LSD from saline, with no dose of *S*(+)-MDMA substituting in either sex, and only high doses of *R*(–)-MDMA producing partial substitution in females [72]. Moreover, 5-HT_2A_R signaling has been implicated in the hyperlocomotive activity and hyperthermic effects induced by *S*(+)-MDMA in male mice [30] and rats [31]. Although our study focused on the potentially stereoisomer-specific effects of MDMA on dendritic structural plasticity in the frontal cortex – and the indirect role of 5-HT_2A_R-dependent signaling in mediating these effects – we cannot exclude the involvement of other GPCRs in behavioral plasticity relevant to abuse liability or therapeutic outcomes, such as social reward learning [73], empathy [74], and prosocial behavior [75]. Potential candidate receptors include 5-HT_1A_R [76], 5-HT_1B_R [75] and 5-HT_2C_R [77]. Similarly, although fluoxetine acts as a highly selective SERT reuptake inhibitor at the tested dose [78], additional studies using alternative pharmacological tools such as escitalopram, or employing transgenic mice targeting the *SERT* (*Slc6a4*) gene [79], are warranted.

HTR is widely considered a rodent behavioral proxy for human psychedelic activity, as it is reliably induced by psychedelic – but not non-psychedelic – 5-HT_2A_R agonists [43], and correlates well with psychedelic effects in humans [80, 81]. Earlier studies demonstrated that the serotonin precursor 5-hydroxytryptophan (5-HTP) [66] and fenfluramine, a serotonin releasing agent, can also induce HTR in rodents [82]. However, the discriminatory power of HTR is excellent for distinguishing between serotonergic modalities, such as blockade of 5-HT reuptake – illustrated by our current findings with fluoxetine – versus direct activation of the 5-HT_2A_R [43, 80]. It has been reported that serotonin itself exhibits psychedelic-like activity in heterologous expression systems [19], and that intracerebroventricular injection of serotonin induces HTR in mice [83]. Additionally, an engineered biosensor based on the structure of 5-HT_2A_R was reported to respond selectively to psychedelic 5-HT_2A_R agonists – but intriguingly, it was also fully activated by endogenous serotonin [84]. Recent work further implicates endogenous serotonin in mediating the HTR induced by the classical psychedelic psilocybin in mice [61]. Supporting this, genetic deletion of SERT significantly reduces HTR induced by psilocybin [79]. Previous studies indicate that the *R*(–)-MDMA isomer is less potent than its mirror image, the *S*(+)-isomer, in promoting 5-HT release in rat whole-brain synaptosome preparations [24], although this stereoselective effect on 5-HT release is not consistently observed in rat hippocampal slices [85]. In the present study, we show that pharmacological blockade of SERT diminishes the HTR elicited by MDMA stereoisomers, which bind to 5-HT_2A_R with low affinity and act as either non-agonists [*R*(–)-MDMA] or weak partial agonists [*S*(+)-MDMA]. Notably, only *S*(+)-MDMA evokes HTR in a sex-biased manner and activates 5-HT_2A_R-dependent signaling through SERT-mediated serotonin efflux. Since classical psychedelics also promote serotonin release in cortical regions [86], these findings raise the alternative, although not mutually exclusive, possibility that endogenous serotonin – rather than direct 5-HT_2A_R agonism – may play a central role in mediating the plasticity-related and therapeutic effects of psychedelics, particularly those that trigger SERT-mediated 5-HT efflux, like DMT [19].

Finally, pharmacological blockade of SERT function – thereby preventing MDMA-induced reverse transport of serotonin – further clarified the relationship between MDMA and its 5-HT_2A_R-dependent effects. Fluoxetine pretreatment abolished *S*(+)-MDMA-induced HTR and IP_1_ accumulation without affecting DOI responses, providing direct evidence that serotonin release – rather than direct receptor activity of this MDMA enantiomer – is responsible for engaging the 5-HT_2A_R in vivo. Intriguingly, previous studies in outbred NIH Swiss mice reported that partial depletion of serotonin via PCPA reduced the HTR induced by *S*(+)-MDMA, but not by *R*(–)-MDMA [60]. These discrepancies may reflect differences between mouse strains, particularly given that the prior work also demonstrated an inverted U-shaped dose-response curve with peak effects occurring at lower doses than those used in the present study. Considering that fluoxetine has also been shown to attenuate many of the subjective effects of MDMA in healthy volunteers [87], collectively, these findings highlight the translational relevance of SERT-dependent and stereoisomer-specific mechanisms underlying MDMA’s effects.

In sum, our results support a model in which MDMA enantiomers – despite differing in their affinity and intrinsic activity at 5-HT_2A_R – induce synaptic and behavioral plasticity through distinct SERT-dependent mechanisms involving serotonin efflux and subsequent activation of cortical 5-HT_2A_Rs. Importantly, these effects are both stereoselective and sex-dependent, underscoring the importance of considering sex as a biological variable in preclinical and clinical studies of entactogens and psychedelics.

## Supplementary information

Supplementary material

## Data Availability

Data will be made available upon request.
