# Supplementary material for: Stereoselective, sex-dependent 5-HT2A receptor modulation of cortical plasticity by MDMA in mice
Source: Neuropsychopharmacology. 2026 Feb 2;51(6):1011–22. doi: 10.1038/s41386-025-02313-x (PMC13125254; doi:10.1038/s41386-025-02313-x)
Supplement: Supplementary file 1 — Supplementary material [file 41386_2025_2313_MOESM1_ESM.pdf]

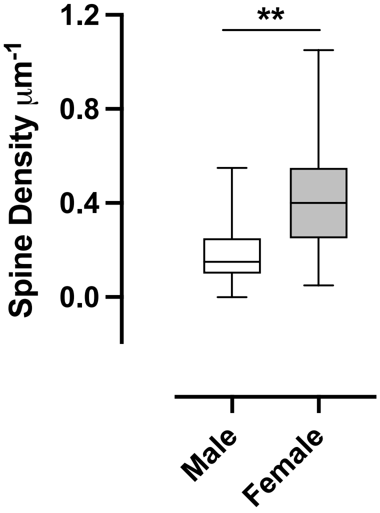

Supplementary Fig. 1. Sex-related differences on dendritic spine density mouse frontal cortex samples. Dendritic spine density in male (n = 28-37 neurons from 3 mice) and female (n = 22-27 neurons from 3 mice) animals. All mice receive vehicle treatment and correspond to those shown in **Fig. 4**. Statistical analysis was performed using Nested *t*-test (**p < 0.001). Box plot presents, in ascending order, the minimum value, first quartile, median, third quartile and maximum value of the sample data, expressed as dendritic spine counts per μm of dendrite.

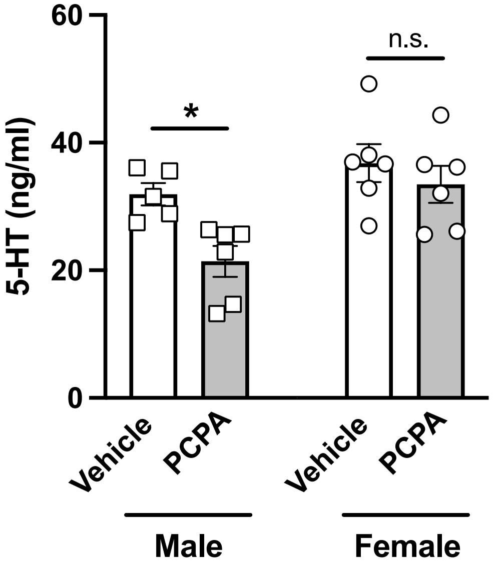

Supplementary Fig. 2. Effect PCPA on 5-HT concentration in the frontal cortex of male and female mice. Mice received (i.p.) PCPA (100 mg/kg) once daily for three consecutive days, and frontal cortex samples of male (5-6 per group) and female (6 per group) mice were collected on the fourth day (Drug: $F[1,19] = 6.89$, $p < 0.05$; sex: $F[1,19] = 10.33$, $p < 0.01$; interaction $F[1,19] = 1.86$, $p > 0.05$). Statistical analysis was performed using two-way ANOVA followed by Bonferroni's post-hoc test (*$p < 0.05$, n.s., not significant). Data show mean ± S.E.M.

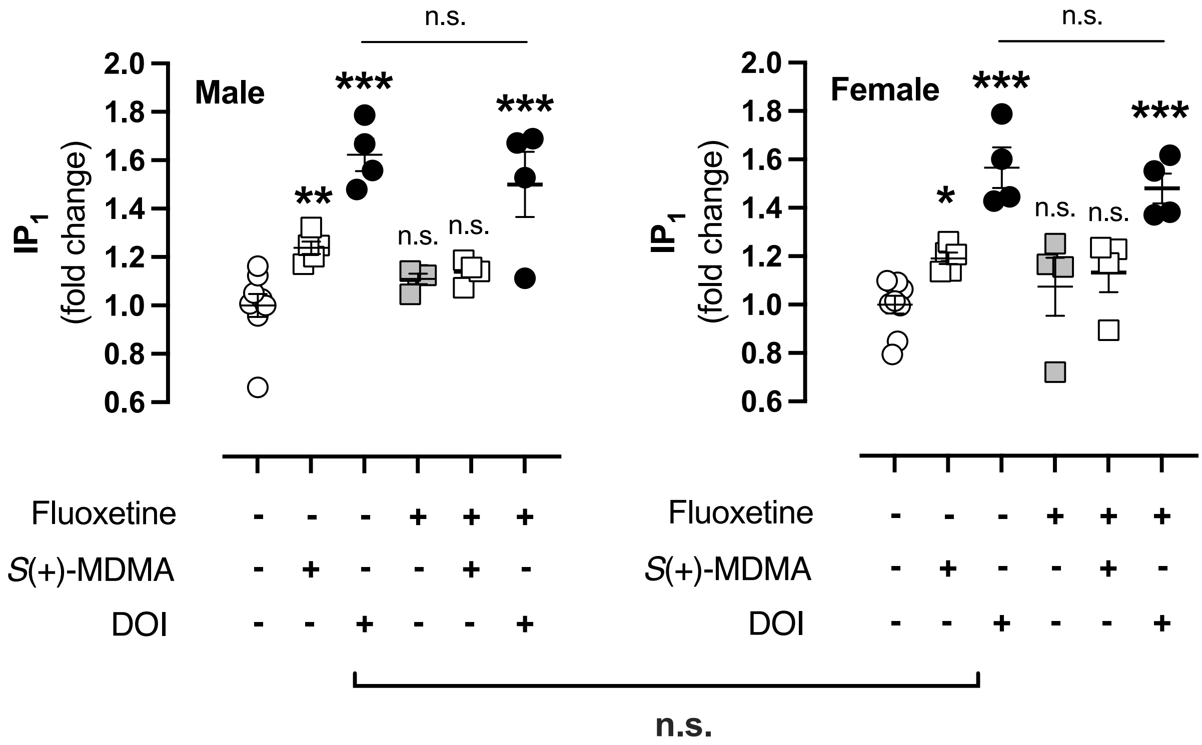

Supplementary Fig. 3. Effect fluoxetine on frontal cortex IP_1 accumulation induced by *S*(+)-MDMA in male and female mice. (**A**,**B**) Effect of pretreatment with fluoxetine (10 mg/kg, i.p.) or vehicle, administered 60 min prior to *S*(+)-MDMA (3 mg/kg, i.p.), DOI (5 mg/kg, i.p.), or vehicle, on IP_1 accumulation in the frontal cortex of male (**A**) and female (**B**) mice (n = 4-9 per group). Samples were collected 60 min after administration of *S*(+)-MDMA, DOI, or vehicle (Drug: $F[5,48] = 28.01$, $p < 0.001$; sex: $F[1,48] = 0.53$, $p > 0.05$; interaction $F[5,48] = 0.07$, $p > 0.05$). Mice that did not receive fluoxetine pretreatment correspond to those shown in **Fig. 3**. Statistical analysis was performed using two-way ANOVA followed by Bonferroni's post-hoc test (*p < 0.05, **p < 0.01, ***p < 0.001, n.s., not significant). Data show mean ± S.E.M.

Supplementary Table 1. Three-way ANOVA analysis of the effect of preadministration of volinanserin on HTR induced by *S*(+)-MDMA or *R*(–)-MDMA in male and female mice.

**Source of variation**	**ANOVA**	**p value**
MDMA	$F[2,71] = 14.13$	$p < 0.001$
Volinanserin	$F[1,71] = 147.8$	$p < 0.001$
Sex	$F[1,71] = 8.21$	$p < 0.001$
MDMA × Volinanserin	$F[2,71] = 7.89$	$p < 0.001$
MDMA × Sex	$F[2,71] = 1.39$	$p > 0.05$
Volinanserin × Sex	$F[1,71] = 6.51$	$p < 0.05$
MDMA × Volinanserin × Sex	$F[2,71] = 2.03$	$p > 0.05$

Supplementary Table 2. Three-way ANOVA analysis of the post-acute effect of *S*(+)-MDMA, and *R*(–)-MDMA on dendritic spine density in the frontal cortex of *5-HT$_{2A}$R-KO* mice and wild-type controls.

**Source of variation**	**ANOVA**	**p value**
MDMA	$F[2,1618] = 44.58$	$p < 0.001$
Genotype	$F[1,1618] = 7.18$	$p < 0.01$
Sex	$F[1,1618] = 144.5$	$p < 0.001$
MDMA × Genotype	$F[2,1618] = 3.68$	$p < 0.05$
MDMA × Sex	$F[2,1618] = 83.10$	$p < 0.001$
Genotype × Sex	$F[1,1618] = 55.48$	$p < 0.001$
MDMA × Genotype × Sex	$F[2,1618] = 18.95$	$p < 0.001$
